# Daesiho-Tang Is an Effective Herbal Formulation in Attenuation of Obesity in Mice through Alteration of Gene Expression and Modulation of Intestinal Microbiota

**DOI:** 10.1371/journal.pone.0165483

**Published:** 2016-11-03

**Authors:** Ahtesham Hussain, Mukesh Kumar Yadav, Shambhunath Bose, Jing-Hua Wang, Dongwoo Lim, Yun-Kyung Song, Seong-Gyu Ko, Hojun Kim

**Affiliations:** 1 Department of Rehabilitation Medicine of Korean Medicine, Dongguk University, Goyang, Republic of Korea; 2 Department of Otorhinolaryngology-Head and Neck Surgery, Korea University College of Medicine, Seoul, Republic of Korea; 3 Institute for Medical Device Clinical Trials, Korea University College of Medicine, Seoul, Republic of Korea; 4 Applied Surface Technology Inc., 11th Floor, Bldg. A, Advance Institutes of Convergence Technology, Yeongtong-gu, Suwon-si, Gyeonggi-do, 16229, Republic of Korea; 5 Key Laboratory of Xin’an Medicine, Ministry of Education, Anhui University of Traditional Chinese Medicine, Meishan Road 103, Hefei, Anhui Province, People’s Republic of China; 6 Department of pathology, College of Korean Medicine, Dongguk University, Goyang, Republic of Korea; 7 Department of Korean Rehabilitation Medicine, College of Korean Medicine, Gachon University, Incheon, Republic of Korea; 8 Department of Preventive Medicine, College of Korean Medicine, Kyunghee University, Seoul, Republic of Korea; Brown University Warren Alpert Medical School, UNITED STATES

## Abstract

**Background:**

Obesity has become a major global health challenge due to its increasing prevalence, and the associated health risk. It is the main cause of various metabolic diseases including diabetes, hypertension, cardiovascular disease, stroke and certain forms of cancer.

**Methods and Results:**

In the present study we evaluated the anti-obesity property of Daesiho-tang (DSHT), an herbal medicine, using high fat diet (HFD)-induced obese mice as a model. Our results showed that DSHT ameliorated body weight gain, decreased total body fat, regulated expression of leptin and adiponectin genes of adipose tissue and exerted an anti-diabetic effect by attenuating fasting glucose level and serum insulin level in HFD-fed animals. In addition, DSHT-treatment significantly reduced total cholesterol (TC), triglycerides (TG) and increased high density lipoprotein-cholesterol (HDL), glutamic pyruvic transaminase (GPT) and glutamic oxaloacetic transaminase (GOT) levels in serum and reduced deposition of fat droplets in liver. DSHT treatment resulted in significantly increased relative abundance of bacteria including *Bacteroidetes*, Bacteroidetes/Firmicutes ratio, *Akkermansia Bifidobacterium*., *Lactobacillus*, and decreased the level of Firmicutes. Using RT2 profiler PCR array, 39 (46%) genes were found to be differentially expressed in HFD-fed mice compared to normal control. However, normal gene expressions were restored in 36 (92%) genes of HFD-fed mice, when co-exposed to DSHT.

**Conclusion/Major Findings:**

The results of this study demonstrated that DSHT is an effective herbal formulation in attenuation of obesity in HFD-fed mice through alteration of gene expressions and modulation of intestinal microbiota.

## Introduction

Obesity, a common metabolic disease, has now become a major global health challenge due to its increasing prevalence, and the associated health risk [1]. Worldwide, approximately 600 million individuals are suffering from obesity [2] and in USA alone, more than 35% of men and women are obese, which has now outnumbered the total overweight individuals [3]. Obesity is associated with various complication and diseases including diabetes, hypertension, cardiovascular disease, stroke and certain forms of cancer [4].

In obesity, increased proliferation and/or differentiation of adipocyte precursor cells has been reported, which facilitates the accumulation of excessive liver fat and elevates lipid concentrations in blood [5]. Increased adipogenesis accompanied by adipocyte differentiation is regulated by a complex network of genes [6]. Accumulating evidence suggests that the gut microbiota also play an important role in human obesity by influencing energy extraction, inflammation, and neuroendocrine secretions [7]. These microbial communities are predominantly represented by two phyla; Firmicutes (includes *Ruminococcus*, *Clostridium*, *Eubacterium* and *Lactobacillus*) and Bacteroidetes (includes *Prevotella*, *Bacteroides*) with significant numbers of *Actinobacteria* (*Bifidobacterium*) and *Proteobacteria* members [8].

Various drugs including orlistat are used for the treatment and prevention of obesity. Orlistat, is a semisynthetic hydrogenated derivative of lipstatin, a natural compound that is produced by the Actinobacterium *Streptomyces toxytricini*. This drug is a potent and selective inhibitor of the gastric and pancreatic lipases, the enzymes playing a key role in the digestion of dietary fat [9, 10] The inhibitory action of orlistat is mediated via covalent binding to the serine residue of the active site of the above mentioned enzymes, thus preventing triglyceride digestion and body weight gain [11]. Other anti-obesity drugs either suppress food-intake by regulating the central nervous system, or inhibit the absorption of specific components in the food [12]. However, these anti-obesity drugs cause manifest various side-effects, including gastro-intestinal discomfort, insomnia, headaches, flatulence and diarrhea [12]. Because of the high prevalence of obesity and unavoidable side-effects of the currently available anti-obesity drugs, the development of herbal medicinal products for treatment of obesity has become a global interest.

DSHT, an important herbal formulation in traditional eastern Asian medicine, is known for anti-diabetic [13], anti-hepatotoxic [14], anti-hypertensive and arterial contraction properties [15], protective effect on hypoxic E18 cortical neuroblast [16] DSHT also showed preventive and therapeutic effects against gallstone formation [17]. However, the anti-obesity activity of this herbal medicine has not yet been studied.

The current study was conducted in-order to evaluate the anti-obesity property of DSHT using high fat diet (HFD) fed obese mice as a model. We also investigated the mode of action of this drug, especially emphasizing its impact on the distribution of gut microbiota and influence on expression of genes playing a key role in the cholesterol and lipid metabolism.

## Material and Methods

### Herbal preparation

The herbal formula DSHT (Daesiho-tang) was procured from Hanpoong pharmaceutical (Seoul, Republic of Korea). It is a combination of eight medicinal herbs; the names and the composition are given in S1 Table. For extraction of DSHT, chopped herbs were mixed with 10 times volume of water and incubated at 100°C for 4 h. After incubation the extract was filtered and freeze dried for 3 days to make dry powder.

### Ethical statement

The animals used in this study were housed and cared in accordance to the National Institute of Health (NIH) guidelines for the care and use of laboratory animals [18]. The experimental protocol was approved by Institute Animal Ethical Committee, Dongguk University, Republic of Korea (No: IACUC 201409111). Animals were anesthetized with a combination of Zoletil (tiletamine-zolazepam, Virbac, Carros, France) and Rompun (xylazine-hydrochloride, Bayer, Leverkusen, Germany) (1:1, v/v).

### Animal experiments and sample collection

Six-week old, male C57BL/6 mice were purchased from Koatech (Pyeongtaek, Republic of Korea). The animals were acclimatized for 4 weeks prior to the experiments. All animals were kept at a constant temperature of 21°C, relative humidity of 55% and under a 12 h light-dark cycle. The animals were maintained on HFD except those who were segregated to serve as normal control (provided with a normal chow diet). After four weeks, animals were divided into four groups, a normal chow diet group as stated above (*n* = 7), HFD group (*n* = 7), and a HFD treated with either DSHT (700 mg/kg/day orally; *n* = 7; DSHT), or orlistat (10mg/kg body weight orally *n* = 7). The normal chow diet was comprised of food with 12.7% of the calories being derived from fat. While, the HFD consisted of food with 60% of the calories being contributed by fat. Body weights of animals were measured weekly. The food intake was measured two times per week during experiment period. Food efficiency ratio was calculated as total body weight gain divided by total food intake (g/g) [19]. Mice were maintained on the treatment for 12 weeks, and were then subjected to oral glucose tolerance test following a 16 h fasting as described below. Finally, the animals were sacrificed under anesthesia as stated above. Blood samples were collected before the animals were sacrificed for the measurements of serum biochemical parameters and insulin level. Tissues used for gene expression analysis were removed, immediately transferred into RNAlater RNA stabilization reagent (QIAGEN, Valencia, CA, USA) and stored at -80°C until needed. For histological analysis, the excised tissues were washed rapidly, fixed in 10% neutral buffered formaldehyde and stored until used.

### Serum biochemistry analysis

After collection, the blood samples were kept at room temperature for 1h to allow clotting, and then centrifuged for 15 min at 3000 rpm. The serum was separated and stored at -80°C until used. The high density lipoprotein-cholesterol (HDL), triglycerides (TG) and total cholesterol (TC) levels were measured using commercial kits (Asan pharmaceutical, Seoul, Republic of Korea) as per kit manufacturer’s instructions. Serum insulin level in the oral glucose tolerance test was determined by enzyme-linked immunosorbent assay using a commercial kit (Mercodia AB, Uppsala, Sweden) following the kit manufacturer’s protocol.

### Oral glucose tolerance test (OGTT)

After the desired treatments, OGTT was performed following a 16 h fasting of the animals. Glucose dissolved in water was administered orally through gavage at a dose of 1 g/kg body weight and OGTT was performed at every 30 min interval (30, 60, 90 and 120 min) after administration of glucose. Blood was collected from the tail vein, serum was separated as described above, and the glucose concentrations of the samples were determined using a blood glucose meter (Accu-Check Advantage, Roche, Charles Avenue, West Sussex, UK).

### Histological analysis of liver

Liver tissues fixed in 10% neutral buffered formaldehyde were sliced (5 μm in thickness) and trimmed transversely into serial sections using a cryo-microtome (LEICA Microsystem, Wetzlar, Germany). Sections were subsequently deparaffinized in xylene, rehydrated through an ascending ethanol series and stained with haematoxylin-eosin (HE) for 1 min and then with oil red O solution (Cayman Chemical, Ann Arbor, MI, USA) for 15 min at 60°C. Mounting was performed with xylene-based media. The slides were observed under an Olympus BX 61 microscope (Olympus Tokyo, Japan) at 200X magnification and images were acquired using Olympus DP70 digital camera (Olympus).

### Measurement of expression of leptin and adiponectin genes in fat tissue

Quantitative real time-PCR (qRT-PCR) was performed to determine the expression of leptin and adiponectin genes in fat tissue. Briefly, total RNA was isolated from the adipose tissue stored in RNAlater RNA stabilization reagent (QIAGEN) using TRIsure reagent (Bioline, Taunton, MA, USA). The RNA samples were quantified and their purity was checked by measurement of optical densities at 260 and 280 nm. The isolated RNA was reverse transcribed using an AccuPower^®^ RT PreMix kit (Bioneer, Daejeon, Republic of Korea) and oligo dt primers (Invitrogen, Carlsbad, CA, USA) in accordance to the kit manufacturer’s protocol. qPCR reaction was performed in a total volume of 20μL, containing 10μL SYBR Green master mix, 1 μg of cDNA, and gene-specific primers (10 pmol for each, S2 Table) using a Light Cycler instrument (Roche, Indianapolis, ID, USA). A dedicated Light Cycler software (Roche Applied Science) was employed for quantification of relative gene expression as 2^-Δct^ using β-actin as a housekeeping gene (^Δ^Ct = Ct_target gene_—Ct_β-actin_) [20]. The results are expressed as normalized fold values relative to the control.

### Microbial analysis of mouse stool using polymerase chain reaction-denaturing gradient gel electrophoresis (PCR-DGGE)

After collection, stools were stored at -80°C until analyzed. Genomic DNA was isolated from the stool using QIAamp stool DNA mini kit (QIAGEN) in accordance to the kit manufacturer’s instructions. The purity and concentration of DNA in the samples were determined using a NanoDrop^™^ (Thermo scientific, Wilmington, DE, USA), and the quality of DNA was further assessed by gel-electrophoresis. The extracted DNA was PCR amplified using universal primers for the bacterial 16S rRNA gene (S2 Table). The first PCR products were separated on 1% agarose gel, stained with ethidium bromide (Etbr, Bio-Rad, Hercules, CA, USA) and 1.5 kb DNA bands were gel-eluted using an Accuprep gel purification kit (QIAGEN). The second round of PCR amplification was performed for the V3 region from 16S rDNA using the primers 314f-GC and 518r (S2 Table) as previously described [17]. DGGE of the second PCR products was carried out using a D Code universal mutation detection system (Bio-Rad, Hercules CA, USA). Briefly, 15 μL of the samples were loaded on 40% acrylamide/bis (37.5:1 v/v) gels with denaturing gradient maintained at 30–60% (where 100% represents 7 M urea in combination with 40% (v/v) formamide) in 1xTAE buffer (ViroMed, Seoul, Republic of Korea). Electrophoresis was performed at 80 V and at a temperature of 60°C for 13 h. After staining with Etbr at room temperature for 20 min, the gels were photographed under UV illumination (LAS-3000; Fuji photo film, Tokyo, Japan). Analysis of the band patterns in the images was carried out using Bionumerics 3.0 software (Applied Maths, Sint-Martens-Latem, Belgium). Data were analyzed using an Unweighted Pair-Group Method with Arithmetic mean (UPGMA) clustering procedure based on genetic similarity expressed by the Jaccard coefficient. Principal component analysis (PCA) was also performed on the DGGE profiles.

### Quantification of bacterial abundance in stool samples using qRT-PCR

qRT-PCR of the stool DNA samples was performed in a total reaction mixture volume of 20 μl as described above for qRT-PCR analysis of fat tissue leptin and adiponectin genes. The primers for the bacterial 16S rRNA genes used in this study are shown in S2 Table. The annealing temperature, concentrations of DNA and primers were optimized as previously described [20]. Analyses of the data and determination of c_t_ value were carried out using Light Cycler software (Roche Applied Science). The relative quantification of bacteria DNA was performed using 2^-Δct^ method, and the normalized fold values relative to the normal group were calculated.

### Gene expression profiling of adipose tissue by RT2-PCR array

Total RNA was isolated from adipose tissues stored in RNAlater RNA stabilization reagent (QIAGEN) using TRIZOL (Invitrogen) according to the manufacturer’s instructions. The extracted RNA was reversed transcribed using a similar method as described above for qRT-PCR analysis of fat tissue leptin and adiponectin genes. Expression of genes involved in lipid and cholesterol metabolism was analyzed by PCR array using a 96-well Human Lipoprotein Signaling & Cholesterol Metabolism RT2 Profiler PCR Array Kit (PAMM-080ZC-2, SABiosciences, Valencia, CA, USA) and a LightCycler 480 PCR system (Roche, Basel, Switzerland) according to the kit manufacturer's instructions. The data were analyzed using SDS Software 2.3 and web-based programs at www.SABiosciences.com/pcrarraydataanalysis.php (SABiosciences). Gene expression was normalized to the mean of all house-keeping genes in the array. Gene ontology (GO) and Kyoto Encyclopedia of Genes and Genomes (KEGG) pathway analysis of the differentially expressed genes were determined using STRING version 10 [21].

### Confirmative gene expression study using qRT-PCR

Seven differentially expressed genes in the RT2 array were further verified using qRT-PCR. The analysis was performed as described above for the qRT-PCR of fat tissue leptin and adiponectin genes using the primers shown in S2 Table.

### Statistical analyses

The values are expressed as mean ± SD. The statistical package for social science (SPSS) software program (version 17.0; SPSS, Chicago, IL, USA) was applied for data analyses. The mean value differences were assessed using Student’s *t*-test, and the statistical significance was set at a *p*-value of less than 0.05.

## Results

### DSHT treatment attenuated total body weight, liver weight, total and intestinal fat in HFD-induced obese mice

**Fig 1:**
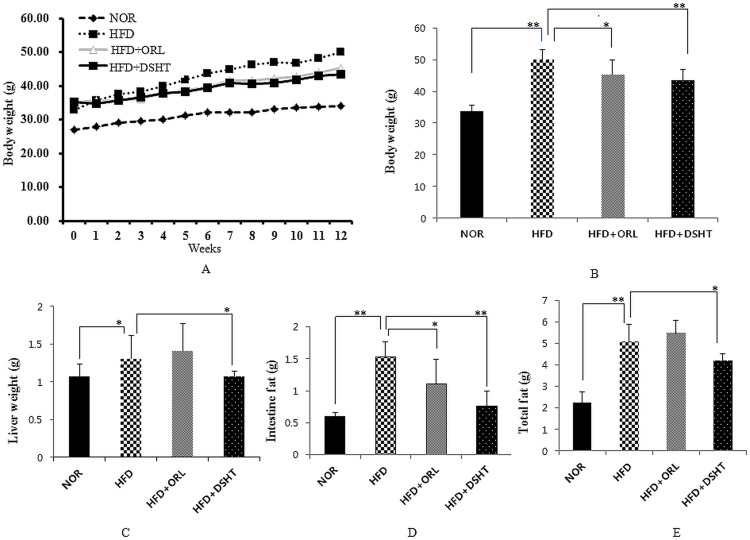
Effect of DSHT on total body weight (gain), liver weight, intestinal and total fat of HFD-fed C57BL/6 mice. (A) Total body weight of normal (NOR), high fat diet (HFD), orilistat (ORL) or daesiho-tang (DSHT) fed animals measured weekly. (B) The total body weight of NOR, HFD, HFD+ORL and HFD+DSHT fed animals after 12 weeks. (C) The liver weight of NOR, HFD, HFD+ORL and HFD+DSHT diet fed animals. (D) Intestinal fat of NOR, HFD, HFD+ORL and HFD+DSHT fed animals. (E) The total fat of NOR, HFD, HFD+ORL and HFD+DSHT fed animals. Data were represented as mean ± SD (*n* = 7). The results were compared by Student’s *t*-test (** corresponds to *p*<0.005, * corresponds to *p*<0.05). The error bars represent the SD.

Total body weight measured weekly increased at a higher rate in HFD-fed animals, while the body weight of HFD + DSHT-treated animals increased at a lower rate (Fig 1A). At the end of 12th week, the body weight of HFD-fed mice was significantly (p<0.05) higher as compared to that of animals in normal group (NOR), indicating an effective HFD-induced obesity in the mice. However, an exposure of HFD-fed animals to DSHT significantly (p<0.005) attenuated total body weight gain (Fig 1B). Similar results were shown by the positive control group (HFD + ORL). The FER was also significantly higher in HFD-fed mice as compared with NOR group. However, this parameter in HFD-fed animals decreased significantly when treated with either DSHT or ORL (S3 Table).

The liver weight, intestinal fat, and total fat were also significantly (p<0.05) augmented in HFD-fed animals compared to that of NOR group. However, DSHT co-treatment significantly (p<0.05) attenuated the liver weight, intestinal fat, and total fat in the HFD-fed mice (Fig 1C–1E). In contrast, co-exposure of HFD-fed animals to ORL caused a significant (p<0.05) reduction in intestinal fat, but did not produce any significant effect on liver weight and total fat. Collectively, our results indicate that DSHT can effectively prevent HFD-induced body weight gain and fat deposition.

### Effect of DSHT on serum biochemical parameters

**Fig 2:**
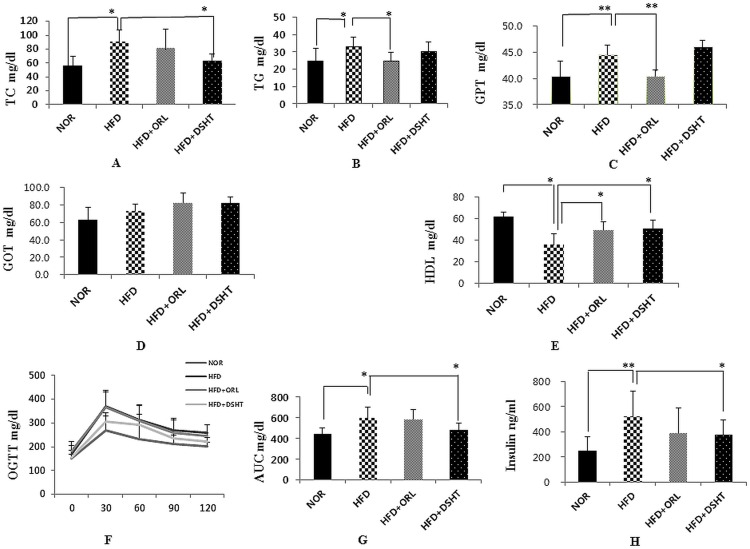
Effect of DSHT on biochemical parameter, glucose homeostasis, and insulin resistance in HFD-fed C57BL/6 mice. (A) The TC levels of normal (NOR), high fat diet (HFD), orilistat (ORL), and daesiho-tang (DSHT) treated animals after 12 weeks. (B) The TG levels of NOR, HFD, HFD+ORL and HFD+DSHT treated animals. (C) The GPT levels of NOR, HFD, HFD+ORL and HFD+DSHT treated animals. (D) The GOT levels of NOR, HFD, HFD+ORL and HFD+DSHT treated animals. (E) The HDL levels of NOR, HFD, HFD+ORL and HFD+DSHT treated animals. (F) Oral Glucose Tolerance test (OGTT) of normal (NOR), high fat diet (HFD), orilistat (ORL) and daesiho-tang (DSHT) fed animals after 12 weeks. (G) Area under curve of NOR, HFD, HFD+ORL and HFD+DSHT fed animals. (H) Fasting insulin of NOR, HFD, HFD+ORL and HFD+DSHT fed animals. Data were represented as mean ± SD (n = 7). The results were compared by Student’s t-test (** corresponds to p<0.005, * corresponds to p<0.05). The error bars represent the SD.

In comparison to NOR, HFD-fed mice showed significantly (p<0.05) higher levels of serum TC, TG, and GPT and significantly (p<0.05) lower content of serum HDL (Fig 2A–2E). However, treatment of HFD-fed mice with DSHT, but not ORL, significantly (p<0.05) attenuated the level of serum TC. The serum TG content was insignificantly and significantly (p<0.05) reduced in HFD+DSHT- and HFD+ORL-treated animals, respectively vs. NOR group. The HDL level was significantly (p<0.05) higher in both HFD+DSHT- and HFD+ORL-treated mice vs. HFD-fed animals No significant differences in the levels of serum GOT and GPT were found between the HFD and HFD + DSHT groups, while HFD + ORL-treated mice showed significantly lower level of serum GPT vs. HFD-fed animals

### DSHT ameliorated glucose homeostasis and insulin resistance

As expected, higher serum glucose levels were observed in HFD-fed animals compared to that of NOR group at all-time points of OGTT (30, 60, 90 and 120 min) (Fig 2F). In parallel, the area under the curve (AUC) of the blood glucose response was also found to be significantly higher in HFD group compared to NOR. However, the animals of HFD + DSHT group, but not HFD + ORL group, showed significantly (p<0.05) lower serum glucose levels at all-time points of OGTT (30–120 min) and AUC of the glucose response compared to that HFD group (Fig 2F & 2G). Following the termination of the OGTT, the serum insulin level was also found to be significantly (p<0.05) elevated in HFD group compared to NOR. However, the serum insulin level was significantly (p<0.05) attenuated in HFD-fed animals when treated with DSHT, but not ORL (Fig 2H)

### DSHT ameliorated HFD-induced histological alterations and fat deposition in liver

**Fig 3:**
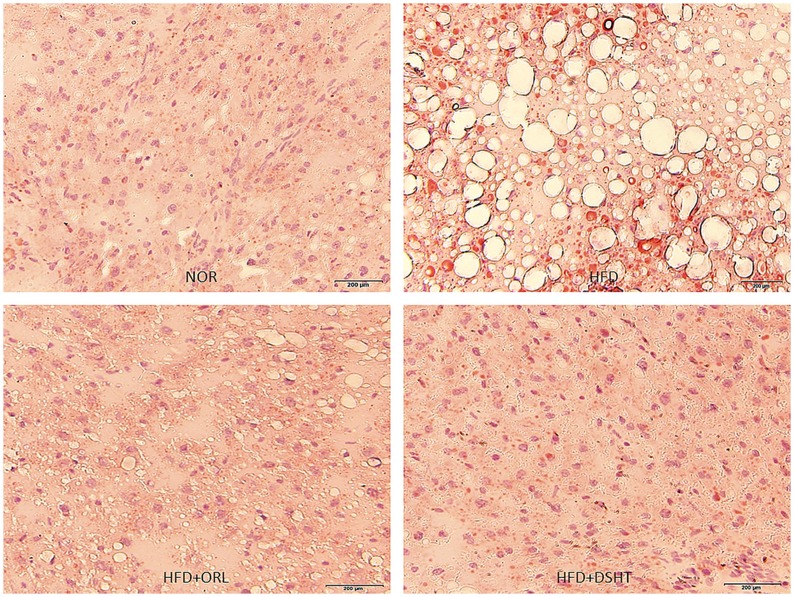
Effect of DSHT on HFD-induced histopathological changes of liver tissue stained with oil red O staining. (A) Representative image of liver tissue of normal control animal (NOR) stained with Oil red O. (B) Representative image of liver tissue of animals treated with HFD. (C) Representative image of liver tissue of animals treated HFD+ORL. (D) Representative image of liver tissue of HFD**+**DSHT treated animals. Pathophysiological examination of the tissue sections was performed under light microscopy with 200x magnification.

Histopathological evaluation of the liver tissue accompanied with oil red O staining demonstrated normal lobular architecture with the appearance of only a few small-sized fat droplets in untreated animals (Fig 3A). In contrast, an aberrant histological structure dominated by substantial deposition of fat in the form of large-sized vacuoles was observed in the liver of HFD-fed animals, indicating the state of fatty liver (Fig 3B). However, the architecture of liver tissue appeared to be normal with minimal or even without deposition of fat droplets in HFD-fed animals treated with DSHT (Fig 3D).a profile quite similar to that shown by ORL (Fig 3C).

### Effect of DSHT on expressions of leptin and adiponectin genes in fat tissues

**Fig 4:**
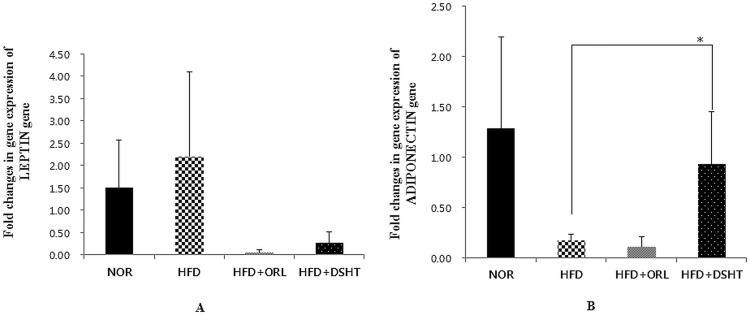
Effect of DSHT on leptin and adiponectin gene expressions in HFD-fed C57BL/6j mice. (A) Folds changes in leptin gene expression in normal (NOR), high fat diet (HFD), orilistat (ORL), and daesiho-tang (DSHT) fed animals after 12 weeks. (B) adiponectin gene expression in NOR, HFD, HFD+ORL and HFD+DSHT treated animals. Data were represented as mean ± SD (*n* = 4). The results were compared by Student’s *t*-test (* corresponds to *p*<0.05). The error bars represent the SD.

To evaluate the changes in gene expression in fat tissues in HFD+DSHT-treated animals, the expression of leptin and adiponectin genes were quantified using real-time RT-PCR. The leptin gene was up-regulated and adiponectin gene was down-regulated in HFD-fed animals compared to NOR (Fig 4). However, DSHT treatment resulted in down-regulation of leptin gene and significant (p<0.05) up-regulation of adiponectin gene in HFD-fed mice. In positive control (HFD + ORL group), also a similar kind of profile was observed for leptin gene expression, however, no increase in adiponectin gene expression was noticed

### DSHT altered gut microbial distribution

**Fig 5:**
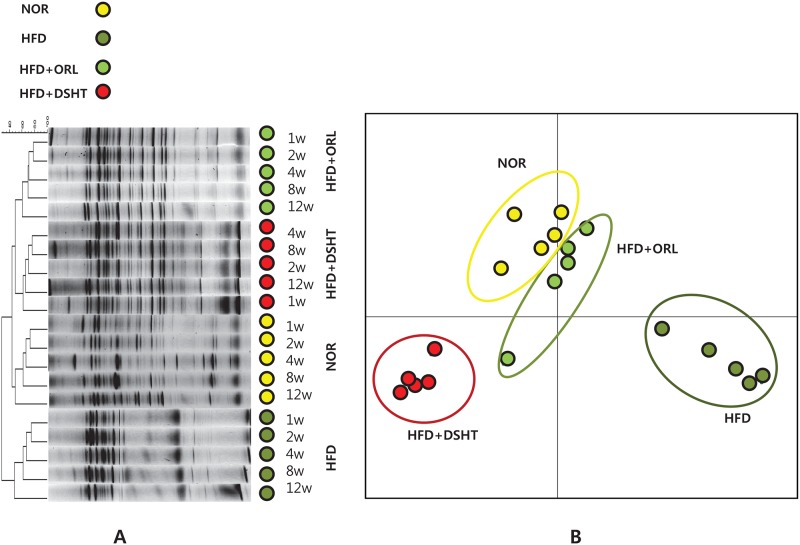
PCR-denaturing gradient gel electrophoresis (DGGE) fingerprinting, and PCA analysis of different microbial flora in mice stool. (A) Banding patters and clustering obtained from PCR-DGGE fingerprinting of mice stool DNA of Normal, HFD, ORL and DSHT. (B) PCA analysis of PCR-DGGE data based on distance matrix showing the similarity between bacterial communities.

HFD Feeding changed the gut floral distribution markedly, which was further altered upon treatment with DSHT or ORL (Fig 5A). The banding patterns obtained in DGGE demonstrated similarities among HFD + DSHT, HFD + ORL, and NOR groups. The PCA analysis also revealed notable differences between the microbial communities of HFD- and HFD + DSHT- treated mice. More specifically, the microbial distribution of animals treated with HFD + DSHT was closely related to that shown by HFD + ORL group (Fig 5B).

**Fig 6:**
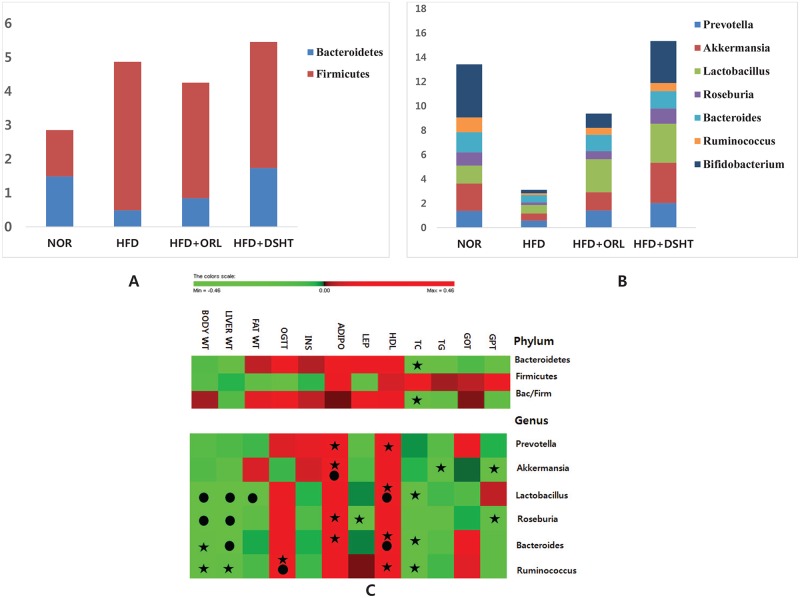
Relative abundances of different microbial flora in mice stool, and their correlations with vital metabolic parameters linked to obesity and liver functions using head-map. (A) The relative abundance of different gut flora was quantified by q-RT-PCR in animals fed on normal diet, High fat diet, HFD+ORL and HFD+DSHT. The relative abundance of Phylum Bacteroidetes decrease and Firmicutes were increased in animals treated with HFD alone. (B) The relative abundance of Genus *Prevotella*, *Akkermansia*, *Lactobacillus*, *Roseburia*, *Bacteroides*, *Ruminococcus* and *Bifidobacterium* were decreased in animals treated with HFD alone. However, the relative abundance of these bacteria was restored to normal in animals treated with DSHT or ORL. (C) Correlations between relative abundance of the gut microbial community and vital metabolic parameters linked to obesity as well as parameters related to liver functions are shown by the heat map. (A symbol of ★ indicates absolute value of Pearson r.>.4; a symbol of ● indicates statistical significance of P<0.05.).

In addition, the population of several gut floral strain, were evaluated using quantitative real time-PCR (Fig 6A and 6B). The relative abundances of phylum Bacteroidetes and genus *Prevotella*, *Lactobacillus*, *Roseburia*, *Bacteroides*, *Ruminococcus*, and *Bifidobacterium* were found to be significantly (p<0.05) lower in HFD-fed animals compared to NOR group. In contrast, the load of phylum Firmicutes was significantly higher in HFD group vs. NOR group. However, the relative abundances of *Roseburia*, *Bacteroides*, *Ruminococcus*, and *Bifidobacterium* in HFD group were significantly (p<0.05) increased upon treatment with either DSHT or ORL. While the exposure of HFD-fed mice to DSHT and ORL caused significant and insignificant increase in *Lactobacillus*, respectively, and insignificant and significant increase in *Prevotella*, respectively. On the other hand, the relative abundance of Bacteroidetes in HFD group was significantly (p<0.05) and insignificantly but prominently increased in response to the treatments with ORL and DSHT, respectively. In contrast, the relative abundance of Firmicutes in HFD-fed animals was insignificantly decreased when exposed to DSHT or ORL. The Bacteroidetes/Firmicutes ratio was insignificantly lower in HFD group vs. NOR group, but increased in both HFD+DSHT and HFD+ORL groups. There was an insignificant, but prominent, decrease in the relative abundance of *Akkermansia* in mice in response to HFD feeding and exposure of the HFD-fed animals to either DSHT or ORL resulted in an insignificant, but definite increase in this bacterial population.

Correlations between relative abundances of the gut microbial communities and vital metabolic parameters linked to obesity as well as parameters related to liver functions are shown by the heat map (Fig 6C). Correlations at each microbial level were determined using two-tailed Pearson’s correlation analysis. At the phylum level, no significant correlation was found between the parameters tested and the relative abundances of Firmicutes or Bacteroidetes or Bacteroidetes/ Firmicutes ratio. However, both Bacteroidetes and Bacteroidetes/ Firmicutes ratio exhibited a definite negative correlation with TC (r = -0.456 and -0.401, respectively). While at the genus level, *Lactobacillus* and *Roseburia* demonstrated significant (p<0.05) negative correlations with the body and liver weights. *Lactobacillus* also showed significant (p<0.05) negative and positive correlations with the total body fat and serum HDL level, respectively, and an insignificant, but obvious negative correlation with serum TC level (r = -0.433). *Roseburia* on the other hand, exhibited certain positive correlation with the gene expression of adiponectin in adipose tissue (r = 0.490). *Prevotella* demonstrated insignificant, but definite positive correlations with both the adipose adiponectin gene expression (r = 0.420) and serum HDL level (r = 0.457). A significant (p<0.05) positive correlation was also found between the adipose adiponectin gene expression and the relative abundance of *Akkermansia*. This genus also showed an insignificant, but a clear negative correlation with serum TG level (r = -0.404). On the other hand, *Ruminococcus* demonstrated a significant (p<0.05) and an insignificant, but obvious (r = 0.493) positive correlations with OGTT and serum HDL level, respectively. This genus also showed insignificant, but prominent negative correlations with body weight (r = -0.475), liver weight (r = -0.422), and serum TC level (r = -0.464). While *Bacteroides* exhibited a significant (p<0.05) negative correlation with liver weight and insignificant, but definite negative correlations with body weight (r = -0.441) and serum TC level (r = -0.456). This genus also showed insignificant but certain positive correlations with adipose adiponectin gene expression (r = 0.455) and serum HDL level (r = 0.511).

### Effect of DSHT on cholesterol and lipid metabolism protein encoding gene expression of C57BL obese mice

**Fig 7:**
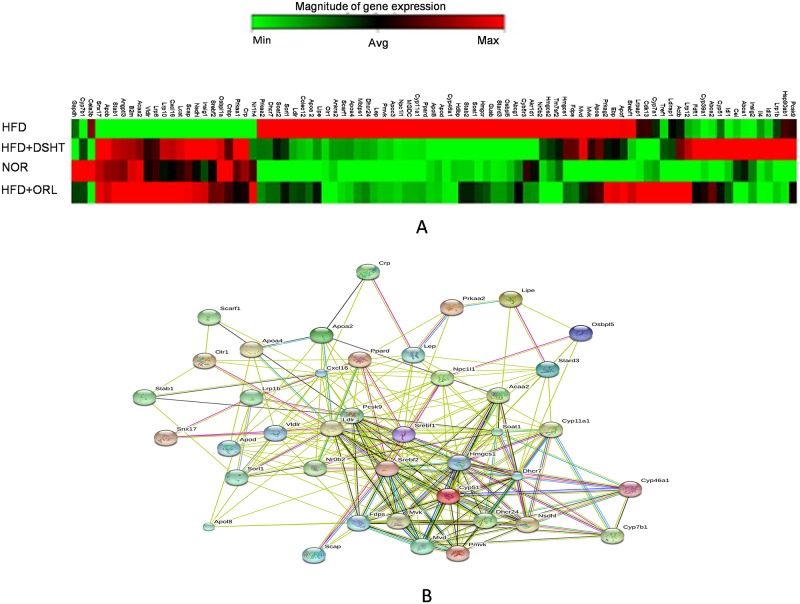
Effect of DSHT on expressions of lipid and cholesterol metabolism genes in HFD-fed c57bl/6j mice. (A) Hierarchical clustering of gene expressions of mouse fed on HFD, DSHT, ORL and NOR detected by RT2 PCR array. The dendrogram at the left provides a measure of relatedness of gene expression in each group. (B) The interconnected pathway of genes differentially expressed in RT2 PCR array.

Expression of genes playing a key role in cholesterol and lipid metabolism was evaluated in the HFD, HFD + ORL and HFD + DSHT groups using RT2 PCR array (Fig 7A). The study revealed that expression of 39 genes was significantly differentially expressed (>2 folds) in HFD-fed animals compared to NOR group. Among them, 29 genes were significantly up-regulated by more than 2 folds, and 10 genes were significantly down-regulated by >2 (0.5) folds (Tables 1 and 2). The GO and KEGG pathway analysis indicated that the genes involved in non-alcoholic fatty liver disease (NAFLD), fat digestion and absorption, insulin signaling pathway, PPAR signaling pathway, AMPK signaling pathway, metabolic pathway, steroid biosynthesis and terpenoid back-bone biosynthesis were up-regulated. And the genes involved in low-protein particle and low-density lipoprotein binding and receptor activity were down-expressed (Fig 7B). More specifically, the genes encoding apolipoproteins (*apoa2*, *apoa4*, *apod*, and *apol8*) and cytochrome P450 family proteins (*cyp11a1*, *cyp461a* and *cyp51*) were up-regulated (>2 folds) in HFD-fed animals compared to NOR, however, expression of these genes in HFD + DSHT-treated animals were decreased compared to HFD group or unchanged vs. NOR. Furthermore except for the *cyp51*, *lrp1b* and *pcsk9* genes, the gene expression patterns in HFD +DSHT-treated mice were similar to those shown by HFD + ORL group. Interestingly, the polytopic transmembrane protein encoding gene Niemann-Pick C1-Like 1 (*npc1l1*), which mediates extracellular sterol transport across the brush border membrane was up-regulated by 10 folds in HFD-fed animals vs. NOR. In contrast, the expression of *npc1l1* was 1 and 1.2 folds (decreased or unchanged) in animals treated with either HFD + DSHT or HFD + ORL, respectively.

**Table 1:** The expression profiles of genes in HFD, HFD + DSHT, or HFD + ORL-treated mice which were significantly up-regulated in HFD-fed animals by 2 folds.

Gene name	Protein	HFD	DSHT	ORL
**Apoa2**	Apolipoprotein A-II	2.3784	1.1173	1.4208
**Apoa4**	Apolipoprotein A-IV	3.7842	1.5692	1.2541
**Apod**	Apolipoprotein D	3.5554	0.6783	0.9571
**Apol8**	Apolipoprotein L 8	4.084	0.8066	1.0994
**Cyp11a1**	Cytochrome P450, family 11, subfamily a, polypeptide 1	6.5432	1.2483	1.7613
**Cyp46a1**	Cytochrome P450, family 46, subfamily a, polypeptide 1	6.1475	0.9461	1.0767
**Cyp51**	Cytochrome P450, family 51	2	4.5631	2.6882
**Dhcr24**	24-dehydrocholesterol reductase	2.514	1.2397	1.3535
**Dhcr7**	7-dehydrocholesterol reductase	4.9246	2.042	2.1535
**Fdps**	Farnesyl diphosphate synthetase	4.7899	3.605	1.9543
**Hmgcs1**	3-hydroxy-3-methylglutaryl-Coenzyme A synthase 1	8.6939	6.2767	3.1529
**Ldlr**	Low density lipoprotein receptor	4.0278	1.4948	2.0658
**Lep**	Leptin	6.5432	1.7777	1.7613
**Lipe**	Lipase, hormone sensitive	2.5315	1.3104	1.7013
**Lrp1b**	Low density lipoprotein-related protein 1B (deleted in tumors)	3.1602	6.9163	1.5547
**Mvd**	Mevalonate (diphospho) decarboxylase	5.0982	4.9588	2.7638
**Mvk**	Mevalonate kinase	3.5064	2.4794	2.4566
**Npc1l1**	NPC1-like 1	10.9283	1.2483	1.7613
**Nr0b2**	Nuclear receptor subfamily 0, group B, member 2	1.9185	1.257	0.9374
**Olr1**	Oxidized low density lipoprotein (lectin-like) receptor 1	5.9381	1.2397	0.8868
**Osbpl5**	Oxysterol binding protein-like 5	4.3469	0.5824	1.5655
**Pcsk9**	Proprotein convertase subtilisin/kexin type 9	2.2346	3.0951	2.1238
**Pmvk**	Phosphomevalonate kinase	3.249	1.1567	1.2368
**Ppard**	Peroxisome proliferator activator receptor delta	3.8106	1.1487	1.4306
**Prkaa2**	Protein kinase, AMP-activated, alpha 2 catalytic subunit	2.0279	1.2834	1.2198
**Scarf1**	Scavenger receptor class F, member 1	2.1287	1.2226	1.0693
**Soat1**	Sterol O-acyltransferase 1	1.9319	0.9138	1.3629
**Sorl1**	Sortilin-related receptor, LDLR class A repeats-containing	2.2501	1.4948	1.5764
**Srebf1**	Sterol regulatory element binding transcription factor 1	2.0279	1.0943	1.9408
**Stard3**	START domain containing 3	2.114	0.9526	1.1783

**Table 2:** The expression profiles of genes in HFD, HFD + DSHT, or HFD + ORL-treated mice which were significantly down-regulated in HFD-fed animals by 2 (0.5) folds.

Gene name	Protein	HFD	DSHT	ORL
**Acaa2**	Acetyl-Coenzyme A acyltransferase 2 (mitochondrial 3-oxoacyl-Coenzyme A thiolase)	0.5905	0.9138	1.0116
**Crp**	C-reactive protein, pentraxin-related	0.4698	1.1329	0.8332
**Cxcl16**	Chemokine (C-X-C motif) ligand 16	0.5905	1.3104	1.4109
**Cyp7b1**	Cytochrome P450, family 7, subfamily b, polypeptide 1	0.3143	0.5625	0.5651
**Nsdhl**	NAD(P) dependent steroid dehydrogenase-like	0.3686	1.6818	1.6096
**Scap**	SREBF chaperone	0.1582	1.434	1.4109
**Snx17**	Sorting nexin 17	0.0272	1.1329	1.0994
**Srebf2**	Sterol regulatory element binding factor 2	0.0188	1.9588	1.4012
**Stab1**	Stabilin 1	0.2588	1.1019	1.2114
**Vldlr**	Very low density lipoprotein receptor	0.028	1.0792	1.8234

While, the *vldlr*, *stab1*, *srebf2*, *snx17*, *scap*, and *nsdh1* genes were down-regulated in HFD-fed animals compared to NOR. However, these genes were significantly up-regulated and/ or maintained a normal gene expression in HFD + DSHT or HFD + ORL-treated animals (Table 2).

### Confirmative Gene expression study using Real-time RT-PCR

**Fig 8:**
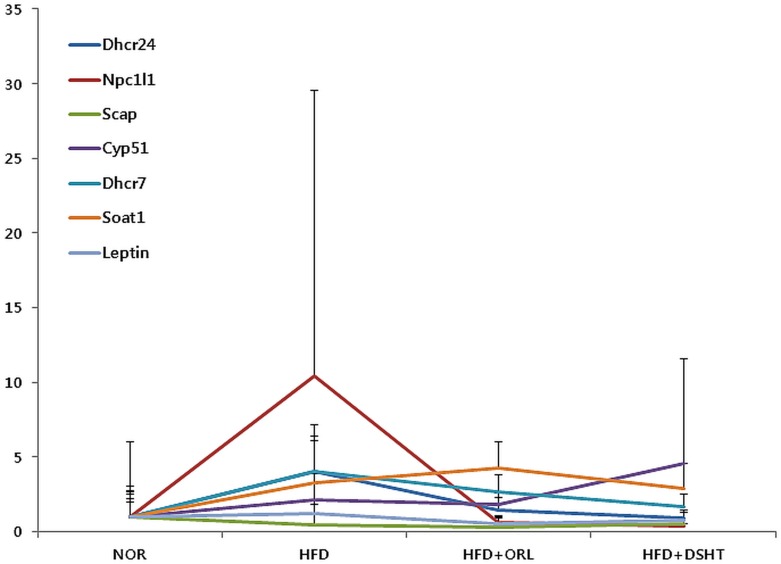
Quantification of gene expression using real-time RT-PCR. To confirm the gene expression of RT array gene expressions, the gene expression of seven differentially expressed genes was evaluated using real-time RT-PCR. Relative gene expression was performed using 2^-Δct^ method and the fold changes in gene expression were normalized with the GAPDH gene. Data were represented as mean ± SD (*n* = 3).

Real-time RT-PCR was used to quantify the expression of seven genes (*dhcr 24*, *npc1l1*, *scap*, *dhcr7*, *leptin*, *soat1* and *cyp51* genes) that were differentially expressed in RT2 PCR array. Real time RT-PCR results were in agreement with RT2 array gene expression data (Fig 8). More specifically, the expressions of *dhcr24*, *dhcr7*, *npc1l1* and *soat1* genes were significantly increased in HFD-fed animals; however, these genes were significantly suppressed in HFD + DSHT-treated animals. Notably, *Scap* gene expression was decreased in HFD-fed mice with respect to NOR, but significantly increased upon treatment with DSHT. *Leptin* gene expression was higher in HFD-fed mice compared to NOR. However, significant reduction in the expression of this gene was observed in HFD + DSHT-treated animals compared to HFD group. The expression of the *cyp51* gene was higher in HFD-fed animals vs. NOR, which was further elevated upon DSHT treatment.

## Discussion

The potential of natural products for treatment of obesity is under exploration, and this may be an excellent alternative strategy for development of future effective and safe anti-obesity drugs. We previously reported on various anti-obesity herbal medicine formulations including *Flos Lonicera* [22], *Rhizoma Atractylodis Macrocephalae* [20], *Bofutsushosan* [23], and *Rehmannia glutinosa* [24]. In this study, we evaluated the anti-obesity property of DSHT using HFD-induced obese mice as a model.

In animals, feeding a HFD induces hypertrophy and hyperplasty of adipocytes, leading to total body weight gain which ultimately results in obesity [25]. In this study, mice fed a HFD gained significantly higher weight than those fed a normal diet indicating that the diet-induced obesity was successfully achieved. However, the mice fed on HFD along with oral supplementation of DSHT did not demonstrate significant weight gain, indicating the anti-obesity impact of DSHT. A similar kind of impact on HFD-fed mice was also shown by ORL, in keeping with recent reports revealing a potent inhibitory effect of orlistat on body weight gain in HFD-induced obese rats and mice [26, 27]. Furthermore, there was significant reduction in liver weight, intestinal fat and total fat in HFD + DSHT-treated mice compared to HFD-fed animals, suggesting that DSHT effectively attenuated proliferation or differentiation of adipose tissues. The total fat mass directly correlated with the body weight and body weight gain; thus, it can be concluded that DSHT suppressed body weight gain by repressing the expansion of adipose tissue mass [28]. In contrast, in HFD group, although ORL significantly decreased the intestinal fat, it failed to produce any significant change in the liver weight and total fat.

It has been found that the serum leptin concentration is directly proportional to the amount of adipose tissue mass [29]. In this study, the leptin protein encoding gene, leptin, was insignificantly, but markedly up-regulated in HFD-fed animals, which however, was down-regulated in both HFD+DSHT and HFD+ORL groups. Leptin is an important regulator of the mass of adipose tissue and body weight, and overexpression of leptin gene has been reported in subcutaneous adipose tissue of massively obese persons [30]. Our findings are also in keeping with a recent study demonstrating depletion in serum leptin concentration in HFD-fed rats in response to the treatment with orlistat [27]. Another obesity-related gene, adiponectin, has been associated with obesity and insulin resistance [31]. In humans, plasma adiponectin concentrations decrease with increasing obesity, and reduced adiponectin concentrations correlate with insulin resistance and hyperinsulinemia [32]. Here expression of the adiponectin gene was lower in HFD group vs. NOR group, but its expression was significantly increased in HFD+DSHT-treated animals, but not in HFD+ORL group.

A previous report has suggested that adipocyte dysfunction-linked obesity imparts insulin resistance and diabetes [33]. In this study, mice fed a HFD showed marked increase in serum insulin and glucose levels and AUC of the glucose response in OGTT. Likewise, elevated serum levels of TC, TG, and GPT and a pronounced decrease in serum HDL level were observed in HFD group compared to NOR group. However, treatment with DSHT, but not ORL, significantly reduced the serum glucose levels and AUC of the glucose response in OGTT in HFD-fed animals, producing a hypoglycemic effect. Importantly, the serum insulin level was also significantly decreased in HFD group in response to treatment with DSHT, but not ORL, endeavoring a protective effect of the proposed herbal formulation on beta cell deterioration [34]. Thus our study suggests the beneficial impact of DSHT on glucose homeostasis and metabolism in HFD-induced obesity. The reasons behind the inability of orlistat in exerting such effects on HFD-fed mice are yet to be clarified. Nevertheless, our findings are in alignment with a previous study revealing that single dose of 120-mg orlistat did not cause any change in postprandial serum glucose, insulin or glucagon-like peptide-1 levels in nondiabetic obese patients [35]. While the levels of aberrant biochemical parameters including TC, TG, HDL, and GPT in HFD-fed mice were either ameliorated or remained unchanged/or normalcy was restored upon treatment with either DSHT or ORL. Based on these, it is conceivable that DSHT and ORL may improve the homeostasis of lipid parameters in HFD-induced obese state. Recent studies have demonstrated that orlistat treatment results in marked improvements in serum and hepatic lipid profiles including TG, TC, low-density lipoprotein cholesterol, and HDL cholesterol in HFD-induced obese rats or mice [26, 27]. Collectively, our findings indicate that DSHT combats body weight gain, reduces total body fat, regulates the expressions of leptin and adiponectin genes in adipose tissue, and exerts an anti-diabetic effect by maintaining glucose homeostasis. The marked improvement in the histopathological characteristics of hepatic tissue in HFD-fed mice by DSHT treatment further supports the anti-obesity impact of DSHT.

Substantial evidence indicate that the gut microbiota play significant roles in diabetes and obesity by influencing energy extraction, inflammation, and neuroendocrine secretions [7, 36–43]; in agreement with our previous findings and other reports revealing that a modulation in the gut bacteria may be linked to obesity [20, 22, 23, 24, 44]. The human gut microbiota encompass more than 1000 phylotypes [43], and it has been estimated that 99% of them are represented by five phyla; Firmicutes, Bacteroidetes, Actinobacteria, Fusobacteria, and Proteobacteria. Among them, Bacteroidetes and Firmicutes account for more than 90% of bacterial population [43]. Growing evidence from clinical studies have revealed that obese subjects with insulin resistance are characterized by an altered composition of gut microbiota, particularly an elevated Firmicutes/Bacteroidetes ratio compared with healthy people [45, 46]. This is further supported by the fact that transplantation of the obese gut microbiota in animals significantly affected the energy harvest of hosts [46]. More specifically, it has been proposed that altered microbiota in obesity modulates intestinal permeability and facilitates metabolic endotoxin secretion that cause chronic low-level inflammation, the hallmarks of insulin resistance and onset of type 2 diabetes [47, 48]. In this study, the PCR-DGGE fingerprinting analysis indicated an alteration in gut microbiota in HFD-fed mice with respect to NOR. While, the banding patterns showed similarities among HFD + DSHT, HFD + ORL, and NOR groups. Therefore, it is conceivable that DSHT treatment probably prevented the aberration of microbial community and restored normalcy in bacterial population in HFD-fed animals. These results were further confirmed by PCA analysis of DGGE fingerprinting. In this analysis, HDF + DSHT-treated animals were clustered close to HFD + ORL and NOR groups, indicating a strong resemblance in the gut microbial communities among these three groups.

To further evaluate the changes in abundance of gut microbiota in HFD-fed mice in response to DSHT treatment, qRT-PCR was performed for quantification of the intestinal flora in stool samples. Our results showed changes in the relative abundances of gut microbial population in response to feeding HFD, mostly in a significant manner. An alteration in gut microbiota at the level of phylum or genus in obesity has been reported in various studies [49, 50]. Based on these evidence, it is conceivable that an imbalance in the abundances of gut bacteria plays a key role in the onset and development of obesity which is likely to be mediated through a number of factors, including boosting of energy harvest from diet, induction of systemic inflammation, and acceleration of fat deposition [51, 52]. Our findings are also in agreement with previous studies where it have been noticed that an increase in the level of Firmicutes and a decrease in the population of Bacteroidetes, an imbalance in Bacteroidetes/Firmicutes ratio, and destabilization of *Akkermansia* population are linked to obesity [48, 53–55]. Similarly, reduced numbers of *Bifidobacterium*, and *Lactobacillus* have been associated with obesity [56, 57].

Accumulating evidence indicate that normalization of the gut microbial population can ameliorate obesity and other associated metabolic alterations and immune dysfunction in animal models [58, 59]. In agreement with this, we found that anti-obesity effects of both DSHT and ORL on HFD-fed mice were associated with restoration of vital gut microbial members as mentioned above. This is also further supported by our findings on correlations between relative abundances of the gut microbial communities and vital metabolic parameters related to obesity. Both Bacteroidetes and Bacteroidetes/Firmicutes ratio showed a definite negative correlation with TC. *Lactobacillus* and *Roseburia* exhibited significant negative correlations with the body and liver weights. *Lactobacillus* also demonstrated a significant negative correlation with the total body fat and an insignificant, but obvious negative correlation with serum TC level. While, *Akkermansia* showed an insignificant, but a prominent negative correlation with serum TG level. *Ruminococcus* revealed insignificant, but clear negative correlations with body weight, liver weight, and serum TC level. *Bacteroides* on the other hand exhibited a significant negative correlation with liver weight and insignificant, but definite negative correlations with body weight and serum TC level. These are in agreement with a recent study which has supported the significance of therapies altering the gut microbiome to regulate body mass, TG and HDL [60]. Additionally, a number of clinical studies have indicated an association between the adiponectin level and obesity-related metabolic dysfunction [61]. More specifically, dysregulation of adipokines has been demonstrated to be associated with obesity, type 2 diabetes, and hypertension [62] These all are in keeping with our findings where significant or marked positive correlations were found between the serum HDL or adiponectin levels or both of these parameters with the relative abundances of *Bacteroides*, *Prevotella*, *Akkermansia*, *Lactobacillus*, *Roseburia*, *and Ruminococcus*. Taking all in consideration, our findings suggest that DSHT or ORL ameliorates HFD-induced obesity partly through restoring the balance in the gut microbial community.

We hypothesize that probable prebiotic effects of DSHT may contribute to the regulation of gut microbial population. It has been found that prebiotics are non-digestible complex carbohydrates that are fermented in the colon, producing energy and short chain fatty acids, and selectively promote the growth of beneficial gut bacteria [63]. Several plants and plant products such as vegetables, root and tuber crops as well as some fruit crops are the well-known sources of prebiotic carbohydrates. So far, fructooligosaccharides (FOS), inulin, and galactooligosaccharides (GOS) from plants are identified as the rich sources of prebiotics. Additionally, the raffinose family of oligosaccharides and resistant starch (the type that is not allowed for absorption in the gastrointestinal tract) has also been found as prebiotic carbohydrates as these molecules are not absorbed in the intestine and facilitate the growth of beneficial gut bacteria [64, 65]. Moreover, some plant cell wall polysaccharides, including xylans and pectins, have also been identified as the chief sources for diverse polysaccharides to generate new prebiotics [66].

Accumulating evidence indicate that multiple indigestible dietary carbohydrates acting as prebiotics can selectively induce the growth of a subset of beneficial gut bacteria and subsequently maintain the homeostasis of gut microbial community as well as the host health [46, 67]. For instance, phylum Bacteroidetes always possess several PULs (polysaccharide utilization loci)-encoded products, which are collectively termed as Sus (starch utilization system)-like systems. These systems encompassing several to decades of enzymes are involved in the degradation of different polysaccharides by targeting specific glycosidic linkages or chemical substituents in the polysaccharides [68]. It has been found that unlike Bacteroidetes, Firmicutes and Actinobacteria express very few carbohydrate-degrading enzymes but enriched with polysaccharide-specific ABC (ATP-binding cassette) transporters which play an important role in the transportation of degraded carbohydrates [69]. This might largely account for the variation of phylum Firmicutes and Actinobacteria in response to the treatment with ginseng polysaccharides as revealed in a previous study [70].

Since DSHT is a multi-herbal medicine, the presence of a variety of prebiotic carbohydrates in this formulation is not an unexpected phenomenon. However, future detailed studies are needed to trace the prebiotics present in DSHT and to elucidate their mode of action in the regulation of gut microbial population in order to validate our hypothesis.

Adipocyte proliferation and differentiation are regulated by a complex network of genes. Accordingly, to identity the potential target genes of DSHT, the expression levels of vital genes regulating lipid and cholesterol metabolism were evaluated in animals treated with HFD either alone or in combination with DSHT or ORL. The results showed that 39 (46%) genes were differentially expressed in HFD-fed mice compared to NOR group. However, no significant changes in gene expression vs. NOR (or restoration of normal gene expression) was shown by in 36 (92%) genes in HFD + DSHT treated animals, indicating that DSHT modulates the vital genes involved in lipid and cholesterol metabolism.

Our study showed the up-regulation of vital lipid and cholesterol metabolism genes- *apoa4*, *ncp1l1*, *srebf1*, *cyp11a1*, *cyp46a* and *cyp51* in HFD-fed mice. The expressions of these genes were either decreased or unchanged in HFD + DSHT-treated animals vs. NOR group. More specifically, the intestinal cholesterol absorption protein encoding gene, *ncp1l1* was up-regulated by >10 folds in HFD-fed mice, however, its expression was decreased to 1-fold in HFD + DSHT-treated animals vs. NOR group. It has been found that *ncp1l1* protein plays an essential role in intestinal cholesterol absorption [71]. Growing evidence strongly suggests that *ncp1l1* inhibition have beneficial impact on the components of metabolic syndrome, such as obesity, insulin resistance, fatty liver, in addition to atherosclerosis [72]. While, the *srebf1* gene encodes the sterol regulatory element binding factor 1 that controls the expression of fatty acid, phospholipid, and triglyceride biosynthetic genes, and the increased expression of *srebf1* was found to be related to obesity [73].

In contrast, the expressions of *vldlr*, *stab1*, and *srebp2* genes were suppressed in HFD-fed animals but restored to normal when treated with DSHT. The *stab1* gene encodes stabilin-1 protein which is involved in the complex physiological clearance processes, up-taking and degradation of the unwanted self-molecules and routing of endogenous and exogenous endocytosis ligands to the secretory pathways [74]. *srebp (sterol-regulatory element-binding protein*)*2* is another important gene down-regulated in HFD-fed mice, but restored to 1.9 fold in HFD + DSHT-treated animals. It has been found that *srebp* is a key regulator of cholesterol homoeostasis, and the reduced expression of this gene may lead to decreased transcriptional activation of the LDL receptor-gene and elevated plasma TC level [75]. Collectively, our gene expression results signify that DSHT treatment regulates the expression of genes involved in NAFLD, fat digestion and absorption, insulin signaling pathway, PPAR signaling pathway, AMPK signaling pathway, metabolic pathway, steroid biosynthesis, terpenoid back-bone biosynthesis, low-protein partical and low-density lipoprotein binding and receptor activity. Probably, the anti-obesity effect of DSHT may be, at least in part, due to the regulation of vital genes involved in cholesterol and lipid metabolism. Notably, we also found that the food efficiency ratio, which was higher in HFD group vs. NOR group, decreased significantly in response to the treatment with either DSHT. Further studies are needed to elucidate whether the altered expression of the above mentioned genes are linked to this effect of DSHT.

## Conclusion

The results of this study demonstrate that DSHT is an effective anti-obesity herbal formulation against HFD-induced obesity. DSHT attenuates obesity in mice through the alteration of gene expressions and modulation of intestinal microbiota. In HDF-fed animals, this formulation reduced the fat deposition, TC, TG, and fasting glucose levels, AUC of the blood glucose response in OGTT, and elevated the HDL level. DSHT also regulates the expression of adipose- related genes, which in turn probably modulates the distribution of the intestinal flora and restores the relative abundances of vital bacteria including *Lactobacillus*, *Akkermansia*, *Bifidobacterium* as well as Bacteroidetes/Firmicutes ratio, and prevents the imbalance of gut microbiota in obese mice. Furthermore, it also regulates a number of vital genes involved in cholesterol and lipid metabolism.

## Supporting Information

S1 Table Composition of DSHT. (DOCX)

S2 Table List of primers used in this study. (DOCX)

S3 Table Food intake and food efficiency ratio. (DOCX)
