# Supplementary material for: Daesiho-Tang Is an Effective Herbal Formulation in Attenuation of Obesity in Mice through Alteration of Gene Expression and Modulation of Intestinal Microbiota
Source: PLoS One. 2016 Nov 3;11(11):e0165483. doi: 10.1371/journal.pone.0165483 (PMC5094769; doi:10.1371/journal.pone.0165483)
Supplement: S1 Table — (DOCX) [file pone.0165483.s001.docx]

**S1 Table** . Composition of DSHT

| Pharmacognostic name | Dried weight (g) |
| --- | --- |
| *Bupleuri radix* | 2.0 |
| *Pinelliae rhizome* | 1.33 |
| *Zingiberis rhizome* | 1.67 |
| *Scutellariae radix* | 1.00 |
| *Paeoniae radix* | 1.00 |
| *Zizyphus fructus* | 1.00 |
| *Ponciri fructus* | 0.67 |
| *Rhei undulati rhizome* | 0.67 |
