# Supplementary material for: Daesiho-Tang Is an Effective Herbal Formulation in Attenuation of Obesity in Mice through Alteration of Gene Expression and Modulation of Intestinal Microbiota
Source: PLoS One. 2016 Nov 3;11(11):e0165483. doi: 10.1371/journal.pone.0165483 (PMC5094769; doi:10.1371/journal.pone.0165483)
Supplement: S2 Table — (DOCX) [file pone.0165483.s002.docx]

**S2 Table .** List of primers used in this study

| Gene/bacteria | Primer sequences |
| --- | --- |
| Lactobacillus. | 5'-GAG GCA GCA GTA GGG AAT CTT C-3'  5'-GGC CAG TTA CTA CCT CTA TCC TTC TTC-3' |
| Bifidobacterium | 5'-CGC GTC TGG TGT CAA AG-3'  5'-CCC CAC ATC CAG CAT CCA-3' |
| Ruminococcus | 5'-GGC GGC CTA CTG GGC TTT-3'  5'-CCA GGT GGA TAA CTT ATT GTG TTA A-3' |
| Akkermansia | 5'-CAG CAC GTG AAG GTG GGG AC-3'  5'-CCT TGC GGT TGG CTT CAG AT-3' |
| Bacteroidetes | 5'-GGA RCA TGT GGT TTA ATT CGA TGA T-3'  5'-AGC TGA CGA CAA CCA TGC AG-3' |
| Firmicutes | 5'-GGA GYA TGT GGT TTA ATT CGA AGC A-3'  5'-AGC TGA CGA CAA CCA TGC AC-3' |
| Prevotella | 5'-CACRGTAAACGATGGATG CC-3'  5'-GGTCGGGTTGCAGACC-3' |
| Roseburia | 5^'^-TACTGCATTGGAAACTGTCG -3^'^  5^'^-CGGCACCGAAGAGCAAT-3^'^ |
| Bacteroides | 5'-GAAGGTCCCCCACATTG-3'  5'-CGCKACTTGGCTGGTTCAG-3' |
| Adiponectin | 5'-TGTTCCTCTTAATCCTGCCCA-3'  5'-CCAACCTGCACAAGTTCCCTT-3' |
| Leptin | 5'-GCCAGGCTGCCAGAATTG-3'  5'-CTGCCCCCCAGTTTGATG-3' |
| Dhcr24 | 5'CGCTGCGAGTCGGAAAGTA3'  5'GTCACCTGACCCATAGACACC3' |
| Npc1l1 | 5'TGTCCCCGCCTATACAATGG3'  5'CCTTGGTGATAGACAGGCTACTG3' |
| Scap | 5'CCGAGCATTCCAACTGGTG3'  5'CCATGTTCGGGAAGTAGGCT3' |
| Soat1 | 5'GAAGGCTCACTCATTTGTCAGA3'  5'GTCTCGGTAAATAAGTGTAGGCG3' |
| Dhcr7 | 5'CAGATTTCTGCCAGGTTATGTGG3'  AGAACCAGGATAAGAGGTAAGCG3' |
| Cyp51 | 5'AACGAAGACCTGAATGCAGAAG3'  5'GTGGGCTATGTTAAGGCCACT3' |
| 16s rRNA | 5’-AGA GTT TGA TCC TGG CTC AG-3’  5’-AAG GAG GTG ATC CAG CC-39’ |
| 314f-GC and 518r | 5’-CGC CCG CCG CGC GCG GCG GGC GGG GCGGGG GCA CGG GGG GCC TAC GGG AGG CAG CAG-3’  5’-ATT ACCGCG GCT GCT GG-3’ |
