# Supplementary material for: Daesiho-Tang Is an Effective Herbal Formulation in Attenuation of Obesity in Mice through Alteration of Gene Expression and Modulation of Intestinal Microbiota
Source: PLoS One. 2016 Nov 3;11(11):e0165483. doi: 10.1371/journal.pone.0165483 (PMC5094769; doi:10.1371/journal.pone.0165483)
Supplement: S3 Table — (DOCX) [file pone.0165483.s003.docx]

**S3 Table. Food intake and food efficiency ratio**.

|  | NOR | HFD | HFD+ORL | HFD+DSHT |
| --- | --- | --- | --- | --- |
| Food intake (g/12 weeks) | 264.9±14.28 | 234.4±10.62^*^ | 212.5±11.56^#^ | 222.8±17.00^#^ |
| Food intake efficiency | 0.025±0.001 | 0.072±0.004^*^ | 0.047±0.003^#^ | 0.036±0.002^#^ |

FER calculate formula: Total body weight gain divided by total food intake

* Significant different from NOR (P<0.05), # Significant Different from HFD Fed (P<0.05)
